# Complete genome of the *Listeria monocytogenes* strain AUF, used as a live listeriosis veterinary vaccine

**DOI:** 10.1038/s41597-024-03440-8

**Published:** 2024-06-17

**Authors:** Valentina A. Feodorova, Sergey S. Zaitsev, Mariya A. Khizhnyakova, Maxim S. Lavrukhin, Yury V. Saltykov, Alexey D. Zaberezhny, Olga S. Larionova

**Affiliations:** 1 Laboratory for Fundamental and Applied Research, Saratov State University of Genetics, Biotechnology and Engineering named after N.I. Vavilov, Saratov, Russia; 2 Department for Microbiology and Biotechnology, Saratov State University of Genetics, Biotechnology and Engineering named after N.I. Vavilov, Saratov, Russia; 3 All-Russian Scientific Research and Technological Institute of Biological Industry, Biocombinat, Moscow Russia

**Keywords:** Live attenuated vaccines, Genome

## Abstract

*Listeria monocytogenes* (Lm) is a highly pathogenic bacterium that can cause listeriosis, a relatively rare food-borne infectious disease that affects farm, domestic, wild animals and humans as well. The infected livestock is the frequent sources of Lm. Vaccination is one of the methods of controlling listeriosis in target farm animals to prevent Lm-associated food contamination. Here we report the complete sequence of the Lm strain AUF attenuated from a fully-virulent Lm strain by ultraviolet irradiation, successfully used since the 1960s as a live whole-cell veterinary vaccine. The *de novo* assembled genome consists of a circular chromosome of 2,942,932 bp length, including more than 2,800 CDSs, 17 pseudogenes, 5 antibiotic resistance genes, and 56/92 virulence genes. Two wild Lm strains, the EGD and the 10403S that is also used in cancer Immunotherapy, were the closest homologs for the Lm strain AUF. Although all three strains belonged to different sequence types (ST), namely ST12, ST85, and ST1538, they were placed in the same genetic lineage II, CC7.

## Abstract (summary)

**Table:** 

**Design Type(s)**	sequence analysis objective • sequence assembly objective• sequence annotation objective • genotyping by high throughput sequencing design
**Measurement Type(s)**	whole genome sequencing • genome assembly • sequence annotation
**Technology Type(s)**	DNA sequencing • whole genome sequencing
**Factor Type(s)**	Organism Strain • live whole cell vaccine genome
**Sample Characteristic(s)**	Listeria monocytogenes

## Background & Summary

*Listeria monocytogenes* (Lm), a highly pathogenic bacterium, is a well-known causative agent of listeriosis. This infection has been proven to be one of the important life-threatening food-borne infections for many species, such as humans, wildlife and farm animals, wild birds, and poultry, being a major concern for both veterinary and public health worldwide^1–7^. Lm pathogen was first recognized as a pathogen in the 1920s during an outbreak in laboratory animals, such as rabbits^1,2,8^. However, research interest in *Listeria* has risen significantly since the 1980s, when the critical role of Lm as the etiologic agent responsible for sporadic cases and numerous outbreaks of human listeriosis was established. These episodes were strictly associated with the consumption of contaminated foods, mainly in Western Europe, Canada, the USA, and Africa^2,3,6,9–15^. Listeriosis is a typical primary zoonotic infection that can be also transmitted vertically in infected pregnant animals and humans, crossing the blood-brain and placental barriers and leading to an invasion into the central nervous system and fetus infection^2,3,5,8,9,14,16–22^. This ubiquitous Gram-positive bacterium can produce in infected mammals a systemic infection leading to bacteremia with serious complications, such as septicemia, meningitis and other central nervous systems (CNS) pathology. Moreover, the pathogen can cause severe focal infections and adverse maternal–neonatal outcomes, including abortion, stillbirth, and preterm birth. Overall, neonatal listeriosis accounts for more than 20% of fatal cases^2,3,5,8,19–22^. Pregnant women, the elderly, and immunocompromised individuals have the highest risk of developing complications and mortality from listeriosis^2,3,5,8,13,19,20^.

Infected farm animals are one of the sources of Lm^5–7,10^. Fecal shedding of Lm was found in dairy cattle (46.3%), beef cattle (30.6%) and sheep herds (14.2%)^10^. Vaccination is considered one of the methods of choice to control listeriosis in target farm animals to prevent the spread of this infection and Lm-associated food contamination, especially for meat and dairy products^4,6,10,17,18,23,24^. Nevertheless, inactivated, live avirulent, or fully virulent Lm strains have been reported to fail in inducing the protective immune response against animal listeriosis^6^.

The attenuated Lm strain AUF has been successfully used for almost 50 years in some regions of the former USSR as a live whole-cell vaccine (LWCV) against listeriosis in farm animals. The Lm strain AUF derives from the fully virulent Lm strain ‘A’ isolated in 1965 from the pathological material of the brain of an ewe with neurolisteriosis^23,24^. The attenuation was done by a combination of 17 repeated exposures of the original strain ‘A’ to Ultraviolet radiation (UVR)^23,24^. The selected Lm strain AUF demonstrated low virulence in animal models (outbred white mice and rabbits) and pronounced immunogenicity in target farm animals (sheep, goats, cows, and pigs)^23,24^. Since the late 1960s, the commercial LWCV based on the Lm strain AUF has been manufactured for the prevention of listeriosis in animal husbandry. However, the genome of the Lm strain AUF has not been sequenced. It is critical to identify the possible genetic markers contributing to the attenuation and residual virulence of this particular Lm strain, which has been used as a commercial LWCV for a long time.

In order to elucidate the basic genomic features of this unique strain, two platforms - Illumina HiSeq 2500 (Illumina Inc., USA) and Nanopore MinION (Oxford Nanopore, UK), intended for short-read and long-read sequencing strategies, respectively, were used in this study in parallel. After quality filtering following the trimming of ‘raw’ massive data, the *de novo* hybrid assembly of the complete genome sequence of the Lm strain AUF represented by a single chromosome 2.94 Gb in size, was successfully obtained. Structural and functional analysis of the Lm strain AUF genome^25^ showed that the assembled complete Lm strain AUF chromosome contained 2,963 genes, including 2,874 CDSs, 17 pseudogenes, 89 RNA genes, 6 rRNA (5 S, 16 S, 23 S), 67 tRNA and 4 non-coding RNA, 5 antibiotic resistance genes (*fosX*, *mprF*, *lin*, *norB* and *sul*), 56 of 92 genes associated with Lm virulence^26,27^ and immunogenicity factors^26,27^, including such key virulence factors as *inlA* and *inlB* which encoded internalin A (InlA) and internalin B (InlB) responsible for binding the host cell receptors E-cadherin and Met, respectively; six genes of the pathogenicity island LIPI-1, *plcA*, *hly*, *mpl*, *actA*, *plcB* and *prfA*, which encoded the transcriptional regulator positive regulator factor A (PrfA) controlling the expression of both inlAB locus and LIPI-1^28–32^ etc.). Additionally, the five-gene stress survival islet (SSI-1, *lmo0444*, *lmo0445*, *lmo0446*, *lmo0447* and *lmo0448*), which is known to confer resistance to environmental stress, such as low pH, high osmolarity, bile and nisin^33^, was annotated in the Lm strain AUF suggesting the possible contribution of these genes to the adaptation of the Lm strain AUF to the relevant conditions. Unfortunately, the culture and sequence of the parental Lm strain ‘A’ isolated more than 50 years ago, is not currently available. In fact, analyses for mutations that might have been induced by the UVR exposure can therefore not be accurately confirmed but rather inferred from closely related fully virulent Lm strains. For this reason, the whole-genome sequence of the Lm strain AUF was compared with nine assembled Lm strains available in GenBank, which had been earlier isolated from different animals with listeriosis, including the referent fully virulent Lm standard strains, the Lm strain FDAARGOS_607, and the Lm strain EGD-e strains approved by the US Food and Drug Administration and the European Consortium, respectively^34–37^. Additionally, the genomes of the Lm strain 10403S and the Lm strain EGD, which have utilized together with the Lm strain EGD-e to analyse the Lm virulence^38^, were selected. We found two groups of loci, which were identified in: (i) both the Lm strain AUF and fully virulent reference strains (virulence (56/73, 76.7%), antibiotic resistance (5/5, 100%), SSI-1 (5/5, 100%), Lm Genomic Islands (2/34, 5.9%), motility (n = 29/31, 93.5%); (ii) only in the fully virulent Lm strains, but not in the Lm strain AUF (virulence (17/73, 23.3%), cadmium resistance (n = 2/2, 100%), SSI-2 (n = 2/2, 100%), Lm Genomic islands (n = 32/34, 94,1%), motility (n = 2/31, 6,5%). Moreover, marked polymorphisms were instantly detected in both nucleotide sequences and allelic profiles of the Lm strain AUF major virulence genes, such as *pfrA*, *hly*, *inlA* and *inlB* which were present in other fully and weakly virulent Lm strains. Importantly, the Lm strain AUF was not absolutely genetically close (3,690 SNPs) to the Lm strain EGD; the Lm strain AUF demonstrated no point mutation in the transcriptional regulator *prfA* typical for the Lm strain EGD compared with the Lm strain EGD-e as G145S^38^. Surprisingly, the Lm strain AUF was genetically close (148 SNPs) to the Lm strain 10403S, which was successfully utilized for the development of attenuated Lm-based cancer vaccine vectors, exploring the Lm unique life cycle and ability to induce robust Cytotoxic T lymphocytes (CTLs) immune responses^39–44^. Nevertheless, contrary to the Lm vectors used in the Lm cancer vaccination platforms which contained truncated virulence genes *prfA*, *actA* and *plcB*^39–44^, both Lm strains, the Lm strain AUF and the Lm strain 10403S, demonstrated the presence of the intact variants of the relevant genes. The Lm strain AUF was markedly distinct from other Lm reference strains, such as the Lm strain EGD-e (31,385 SNPs), the Lm strain NTSN (141,219 SNPs), the Lm strain FDAARGOS_607 (144,871 SNPs), the Lm strain UKVDL9 (178,505 SNPs), the Lm strain UKVDL4 (178,552 SNPs), the Lm strain UKVDL7 (180,314 SNPs), the Lm strain 4/52-1953 (182,819 SNPs) and the Lm strain FSL-J1-158 (196,422 SNPs).

The data presented here is the first report highlighting the genome loci which could be either directly or indirectly involved in the attenuation (LIPI-3, *srtB*, *agrC*, *vip*, *gltB*, *gltA*, *aut_IVb*, metal resistance genes and Lm Genomic Islands-associated genes) and residual virulence (LIPI-1, *plcA*, *hly*, *mpl*, *inlA*, *inlB*, *prfA*, *hly*, *actA* and *plcB*) of the single Lm strain AUF with a long history of application as an effective veterinary LWCV against listeriosis. Our data could also serve as the basis for unravelling the mechanisms of virulence and pathogenicity in Lm. The results of this study will be useful for improving our knowledge of bacterial vaccinology overall and developing of a new generation of vaccines against listeriosis in farm animals. This is critical for animal health and welfare, food safety, and human public health worldwide.

## Methods

### Bacterial strain

The Lm strain AUF was obtained from the Collection of Microorganisms of the Department for Microbiology and Biotechnology, Saratov State University of Genetics, Biotechnology and Engineering named after N.I. Vavilov, Saratov, Russia. The Lm strain AUF was stored in lyophilized form. The Lm strain AUF was routinely cultivated on Tryptone Soy Yeast Extract Agar (TSYE Agar) (Merck, EU) overnight prior to the experiments as described^45,46^.

### DNA Extraction for short & long reads sequencing

Genomic DNA was extracted from the lysate of the Lm strain AUF culture, using a commercial DNA DNeasy Blood & Tissue Kit (Qiagen, Germany) according to the manufacturer’s instructions as described^45,46^. The final DNA concentration was measured using a spectrophotometer from BioRad (Bio-Rad, USA).

### Whole genome sequencing

The isolated DNA from the Lm strain AUF strain was used in two parallel sequencing platforms, Illumina HiSeq 2500 (Illumina Inc., USA) and Nanopore MinION (Oxford Nanopore, UK). For this purpose, the preparation of DNA libraries was conducted using either Nextera XT DNA Library Preparation Kit (Illumina Inc., USA) or Nanopore Kit SQK-LSK109 (Oxford Nanopore, UK), respectively, as we described recently^45,46^. In the first case, the DNA was sequenced commercially as 250-bp single-Read (Illumina Inc., USA) at Genoanalytica LLC (https://www.genoanalytica.ru/, Moscow, Russia). A FLO-MIN-106 R9.4 Flow cell (Oxford Nanopore Technologies, Oxford, UK) was routinely used to perform sequencing with the MinION and the MinKNOW software as recommended (https://nanoporetech.com/).

### Genome assembling and annotation

Unicycler v0.4.9 with default parameters (https://github.com/rrwick/Unicycler) was used to generate a hybrid high-quality *de novo* assembly of the Lm strain AUF strain genome^47^. The annotation of chromosome was performed by the NCBI Prokaryotic Genome Annotation Pipeline (PGAP) with default parameters on the submission portal of NCBI GenBank (https://www.ncbi.nlm.nih.gov/genome/annotation_prok/)^48^. Construction of the phylogenetic tree based on the Lm strain AUF and other *Listeria spp*. strain whole genomes was conducted with the online tool REALPHY 1.13 using default parameters (https://realphy.unibas.ch/realphy/)^49^ and MEGA-7 software^50^ by the maximum likelihood method for the generation of the phylogenetic tree based on the internalin genes. Antibiotic resistance genes were identified using CARD (https://card.mcmaster.ca/analyze/rgi) and the Antibiotic Resistance scheme of the Institute Pasteur Bacterial Isolate Genome Sequence Database (BIGSdb Version 1.42.0) (https://bigsdb.pasteur.fr/listeria/). Comparative analysis of the CDSs in the Lm strain AUF strain relative to the Lm reference strains was performed using «Proteome Comparison Service» tool on The Bacterial and Viral Bioinformatics Resource Center (BV-BRC) platform^51^. Visualization of the linear map of the Lm strain AUF genome was generated using the online tool Proksee (https://proksee.ca/)^52^. Genes encoding virulence factors in the Lm strain AUF and other Lm strains were found using the BIGSdb-Lm database (https://bigsdb.pasteur.fr/listeria/). Identification of allele profiles of the Lm strain AUF and reference Lm strains whole genome and Multilocus sequence typing (MLST) was also performed using the BIGSdb-Lm database (https://bigsdb.pasteur.fr/listeria/). Comparative analysis of pseudogenes in the Lm strain AUF and other Lm strains was performed using BLAST (https://blast.ncbi.nlm.nih.gov/Blast.cgi).

### A panel of Lm reference strains

We compared the annotated completed genomes of ten Lm strains available in the NCBI GenBank (https://www.ncbi.nlm.nih.gov/). The relevant whole genome sequences were downloaded: the Lm strain FDAARGOS_607^53^, the Lm strain EGD-e^54^, the Lm strain EGD^55^, the Lm strain FSL-J1-158^56^, the Lm strain NTSN^57^, the Lm strain UKVDL9^58^, the Lm strain UKVDL4^59^, the Lm strain 4/52-1953^60^, the Lm strain UKVDL7^61^ and the Lm strain 10403S^62^. Additionally, the genomes of 12 strains of other *Listeria spp*. were used for the construction of a phylogenetic tree in order to demonstrate the phylogenetic relationships between the Lm strain AUF and other *Listeria*, such as: the *Listeria grayi* strain NCTC10812^63^, the *Listeria valentina* strain CLIP 2019/00642^64^, the *Listeria kieliensis* strain Kiel-L1^65^, the *Listeria floridensis* strain FSL S10-1187^66^, the *Listeria fleischmannii* strain 1991^67^, the *Listeria rustica* strain FSL W9-0585^68^, the *Listeria newyorkensis* strain CMB191063^69^, the *Listeria cornellensis* strain FSL F6-0969^70^, the *Listeria rocourtiae* strain CECT 7972 Ga0244616_101^71^, the *Listeria booriae* strain FSL A5-0281^72^, the *Listeria riparia* strain FSL S10-1204^70^, the *Listeria phage* strain A118^73^.

## Data Records

The completed whole genome sequence of the Lm strain AUF has been deposited in GenBank with the accession number CP048400.1^47^. All of the reads for the Lm strain AUF genome have been deposited in the NCBI Sequence Read Archive under the accession numbers SRR25210281 for Oxford Nanopore data^74^ and SRR25180708 for Illumina HySeq 2500 data)^75^.

## Technical Validation

Before starting the genome assembly, raw reads were prepared using an automated AfterQC script to remove low-quality reads and Illumina adapters in default settings for short reads, as well as to remove adapters using Filtlong v0.2.1 software. To filter long reads by quality obtained on the Oxford Nanopore platform, we used the Filtlong script (https://github.com/rrwick/Filtlong) with the filter parameters --min_length 1000 --keep_percent 90. Trimming and searching for Nanopore adapter sequences in long reads was performed using the Porechop script according to the recommended parameters (https://github.com/rrwick/Porechop). The *de novo* assembly using both short and long reads, obtained from both platforms, Illumina and Nanopore MinION, generated the Lm strain AUF circular chromosome of 2,942,932 bp in size with a GC content of 37.98%.

**Fig. 1:**
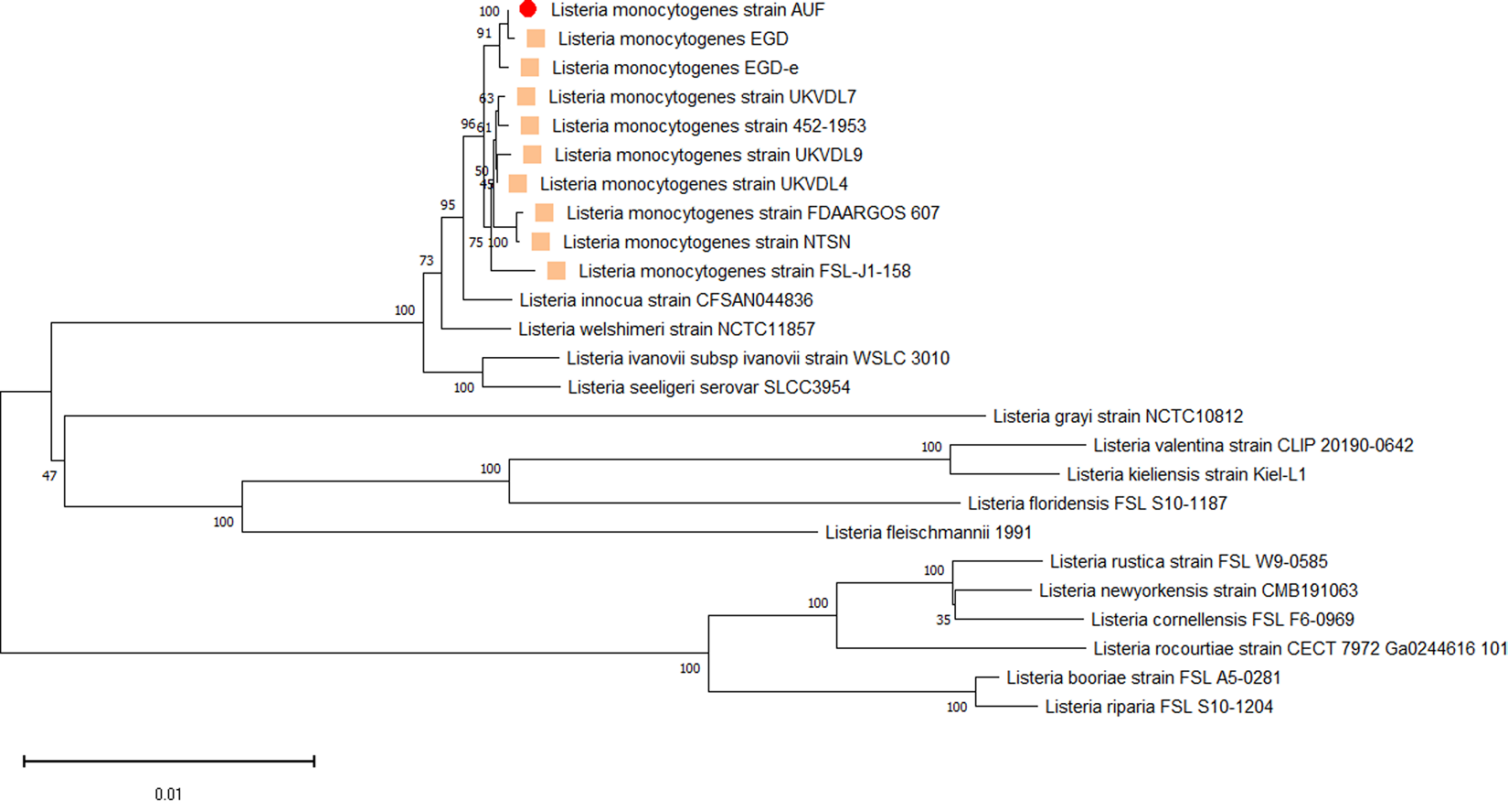
Phylogenetic analysis of the Lm strain AUF using the reference whole genome sequences of Lm and other *Listeria spp*. available in NCBI GenBank (https://ncbi.nlm.nih.gov/). The phylogenetic tree was built using REALPHY 1.13 (https://realphy.unibas.ch/realphy/) and visualized with MEGA-7^30^.

To clarify the taxonomic affiliation of the assembled Lm strain AUF genome to other *Listeria spp*. representatives, we constructed a phylogenetic tree based on the whole genomes available in the NCBI GenBank (https://www.ncbi.nlm.nih.gov/). For this purpose, we selected the reference genomes of 15 different *Listeria spp*. and 9 Lm strains associated with listeriosis in animals. The Lm strain AUF formed a separate branch with representatives of the Lm only, but not with other *Listeria spp*. reference bacteria strains, which proved its genetic affiliation to the indicated Lm species (Fig. 1). Importantly, the Lm strain AUF visualized in a single clade with the fully virulent Lm strain EGD, which was isolated in 1924 in the United Kingdom from a guinea pig infected with biomaterial derived from a rabbit with listeriosis during the disease outbreak in laboratory animals^1,2,8^. The data obtained recognized the EGD as the closest homolog to the Lm strain AUF, in contrast to other Lm representatives, including both the Lm strain EGD-e and the Lm strain FDAARGOS_607 (Fig. 1). The Lm strain EGD-e was assigned as the second homolog for the Lm strain AUF located in the same cluster with the Lm strain AUF and the Lm strain EGD strains (Fig. 1). The Lm strain AUF was also distinguished from the Lm 4/52-1953, had also been isolated on the territory of the former USSR, although a decade earlier^46^. In fact, recently phylogenetic analysis based on whole genomes of Lm strains (n = 257) available in the NCBI GenBank (https://www.ncbi.nlm.nih.gov/) showed that these two Lm strains belonged to different Clusters, the Lm strain AUF to the Cluster II, represented by genetic lineages I and II, whereas the Lm strain 4/52-1953 - to the Cluster I, formed by genetic lineage III only^46^. Interestingly, the Lm strain AUF was phylogenetically closer to the Lm strain 10403S derived from the clinical sample of a human with listeriosis^46^. Both strains, the Lm strain AUF and the Lm strain 10403S, formed a single Cluster II^46^. The *prfA*-, or *act*A- or *plcB*- defective derivatives of the Lm strain 10403S were reported to be safe live attenuated Lm vectors for the development of cancer immunotherapy vaccines^39–44^.

**Fig. 2:**
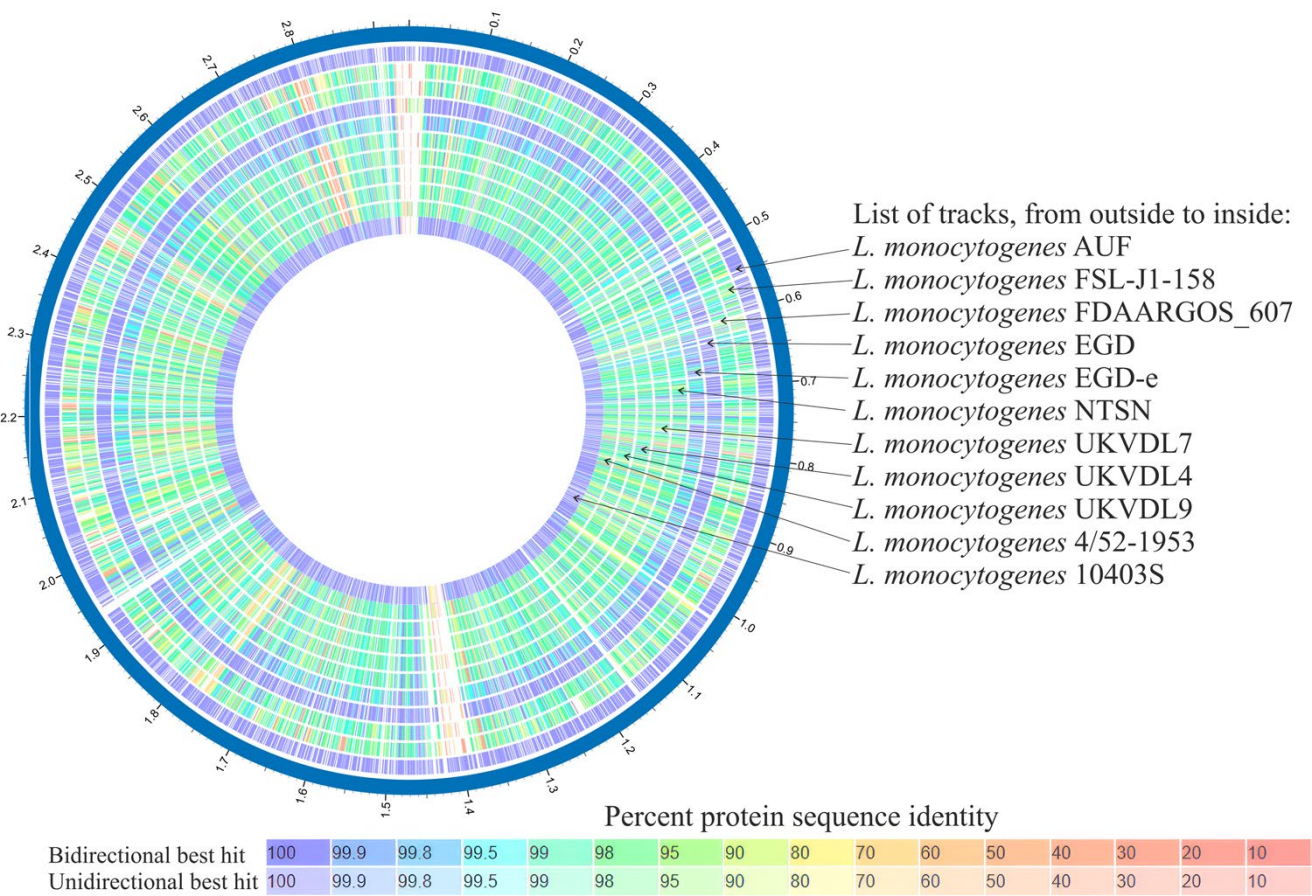
Visualization of the alignment of the coding regions of the Lm strain AUF versus ten reference Lm strains using «Proteome Comparison Service» tool on The Bacterial and Viral Bioinformatics Resource Center (BV-BRC) platform^31^. Protein sequence identity is determined on a colorimetric scale, where purple/blue colors correspond to a higher percentage of identity than orange/red colored regions (based on the Best Bidirectional Hits and Unidirectional best hit comparison algorithms). The white areas represent the absence of coding regions annotated at that location in the chromosome sequence of the respective strain.

Through MLST, based on the sequences of seven housekeeping genes (*abcZ*, *bglA*, *cat*, *dap*, *dat*, *ldh* and *lhkA*), we found that the Lm strain AUF together with the closest homologs, the fully virulent reference strains, the Lm strain EGD, the Lm strain EGD-e and the Lm strain 10403S, belonged to the same genetic lineage II, although these strains sourced from different hosts, such as sheep, rabbit and human, respectively (Table 1). Moreover, the Lm strain AUF, the Lm strain EGD and the Lm strain 10403S corresponded to the identical clonal complex (CC) CC7, contrary to the Lm strain EGD-e which was assigned to CC9. Nevertheless, the Lm strain AUF, the Lm strain EGD and the Lm strain 10403S related to the different sequence types (ST), ST1538, ST12 and ST85, respectively, although all three strains demonstrated six of seven identical alleles (*abcZ*, *bglA*, *cat*, *dap*, *dat*, and *lhkA*) with polymorphism in only a single allele, *ldh* (Table 1). We found only one individual allele, *lhkA*, which was identical for the Lm strain AUF, the Lm strain EGD, the Lm strain 10403S and the Lm strain EGD-e. No identical alleles were revealed between the Lm strain AUF and two ovine fully virulent Lm strains belonging to the genetic lineage I, the Lm FDAARGOS_607 and the Lm strain NTSN, and the ovine, bovine, porcine, equine and caprine Lm strains, the Lm strain UKVDL9, the Lm strain UKVDL4, the Lm strain 4/52-1953, the Lm strain UKVDL7, the Lm strain FSL-J1-158, the Lm strain the Lm strain UKVDL7 and the Lm strain of the genetic lineages III-IV belonging to different STs, ST1194, ST1069, ST201, ST1140 and ST563 of clonal complexes ST1194, ST1069, CC69, CC1070 and ST563 (Table 1). Comparative analysis of the coding regions using BLASTP^51^ proved the marked discrimination of the Lm strain AUF genome from the other ten representative Lm strains (Fig. 2). As expected, the Lm strain AUF showed the highest homology of coding regions with the Lm strain 10403S, the Lm strain EGD, a few somewhat lower one with the Lm strain EGD-e one, and much lower one with other Lm reference strains compared.

**Table 1:** MLST characteristics the Lm strain AUF and Lm reference strains isolated from animals with listeriosis, whose whole genomes are available in the NCBI GenBank (https://www.ncbi.nlm.nih.gov/).

The Lm Strain	NCBI Accession Number	Source	Country	Allele							ST	Clonal complex (CC)	Genetic line (Lineage)	Reference/Source
				*abcZ*	*bglA*	*cat*	*dapE*	*dat*	*ldh*	*lhkA*				
AUF	CP048400.1	Sheep	USSR	**5**	**8**	**5**	**7**	**6**	65	**1**	ST1538	CC7	II	This study
EGD	HG421741.1	Rabbit	Great Britain	**5**	**8**	**5**	**7**	**6**	22	**1**	ST12	СС7	II	^8,27^
10403S	NC_017544.1	Human	USA	**5**	**8**	**5**	**7**	**6**	38	**1**	ST85	CC7	II	^38–44,46,61^
EGD-e	AL591824.1		Great Britain	6	5	6	20	1	4	**1**	ST35	CC9	II	^38,45–48,116–118^
FDAARGOS_607	CP041014.1	Sheep	USA	3	1	1	1	18	28	3	ST449	CC1	I	
FSL-J1-158	CP090057.1	Goat	USA	84	75	92	101	59	227	73	ST563	ST563	IV	^119^
NTSN	CP009897.1	Sheep	China	3	1	1	1	3	1	3	ST1	CC1	I	^22^
UKVDL9	CP065028.1	Sheep	USA	116	33	204	253	34	388	28	ST1194	ST1194	III	^120^
UKVDL4	CP076644.1	Cattle	USA	204	105	194	24	151	145	161	ST1069	ST1069	III	^120^
4/52-1953	CP048401.1	Piglet	USSR	19	17	22	25	15	79	12	ST201	CC69	III	^46^
UKVDL7	CP076669.1	Horse	USA	189	159	99	225	32	355	150	ST1140	CC1070	III	^120^

The identical alleles are highlighted in bold.

Comparative analysis of the whole genomes of the Lm strain AUF versus nine Lm reference strains of zoonotic source on the BV-BRC platform (https://www.bv–brc.org/) showed 100% homology in more than 90% CDSs in the Lm strain EGD strain, approximately 50% in the Lm strain EGD-e and only 12–14% in other Lm genomes (Table 2). Similarly, the highest homology in CDSs ranging from 20–79% demonstrated in the majority of Lm strains except both closest homologs. Also, approximately 200 CDSs were present in only the Lm strain AUF and absent in all other strains except the Lm strain EGD. About 100 CDSs were annotated in the Lm strain AUF but not in the Lm strain EGD^76^. These Lm strain AUF-specific genes were located on the Lm strain AUF chromosome as several compact regions, which were visualized as extended insertions in the Lm strain AUF compared with other reference Lm strains used, including both closest homologs, the Lm strain EGD and the Lm strain EGD-e, and the Lm strain 10403S as well (Fig. 2). The majority of these Lm strain AUF-specific genes encoded uncharacterized hypothetical proteins, *Listeria* CRISPR-associated proteins (Cas1/Cas2/Cas9/Csn2)^77^, phage proteins, Type I restriction-modification system components, and in contrast to some Lm strains, GNAT family Acetyltransferase, Gp45 protein, 4-hydroxy-2-oxoglutarate aldolase (EC 4.1.3.16), 2-dehydro-3-deoxyphosphogluconate aldolase (EC 4.1.2.14), transcriptional regulator, ADP-ribosyl glycohydrolase, putative EsaC protein analog (*Listeria* type 3), cassette chromosome recombinase B, and mobile element proteins. Bacteriophage A118 genes which have been known as *Listeria* phage A118 (NCBI Acc. number: NC_003216.1), and initially hosted in the Lm strain EGD-e^78–81^, were successfully detected in the Lm strain AUF and all other Lm strains used in the current study except the Lm strain EGD^76^. Comparison of the whole genomes of the Lm strain AUF with the Lm strain 10403S sourced from human sample using the similar BV-BRC platform (https://www.bv–brc.org/) demonstrated 100% homology for about 93% CDSs, 80 – 99% identity for slightly more than 3% CDSs and less than 1% in CDSs ranging from 20-79% (Table 2). Only 93 CDSs were annotated in the Lm strain AUF. These were absent in the Lm strain 10403S^76^. Basically, there were the same Lm strain AUF-specific genes which encoded uncharacterized hypothetical proteins and phage proteins^76^.

**Table 2:** Comparative homology in the CDSs of the Lm strain AUF versus the Lm reference strains.

Comparison the Lm strain AUF versus the Lm Strain	Number of CDSs with homology, %						Number of CDSs found in only the AUF strain	
	100.0		80.0–99.0		20.0–79.0			
	Abs.	%	Abs.	%	Abs.	%	Abs.	%
AUF vs EGD	2,608	90.7	204	7.0	20	0.7	97	3.4
AUF vs 10403S	2,722	92.8	98	3.3	18	0.6	93	3.1
AUF vs EGD-e	1,409	49.0	1,325	46.1	41	1.4	155	5.4
AUF vs FDAARGOS_607	428	14.9	2,197	76.4	106	3.7	197	6.9
AUF vs NTSN	420	14.6	2,197	76.4	92	3.2	238	8.3
AUF vs 4/52-1953	348	12.1	2,215	77.0	155	5.4	210	7.3
AUF vs UKVDL9	348	12.1	2,204	76.7	118	4.1	258	9.0
AUF vs UKVDL7	356	12.4	2,190	76.2	150	5.2	232	8.1
AUF vs UKVDL4	345	12.0	2,238	77.9	113	3.9	232	8.1
AUF vs FSL-J1-158	348	12.1	2,215	77.0	155	5.4	210	7.3

Additionally, we found (Table 3) in the Lm strain AUF approximately 60% (56/92) of genes that have been recently recognized as those involved in Lm virulence^26^. This amount was 7-10% less than that revealed in both fully virulent Lm strains of the genetic lineage I, the Lm strain NTSN (62/92, 67.4%) and the Lm strain FDAARGOS_607 (64/92, 69.6%). However, the number of virulence genes in the Lm strain AUF was certainly higher than that in the Lm strains of the genetic lineages III-IV, up to 1.3 - 3.9 times, for instance, in the Lm strain UKVDL7 (14/92, 15.2%) and the Lm strain 4/52-1953 (41/92, 44.6%), respectively. In fact, the Lm strain AUF demonstrated almost similar characteristics in this regard compared with the other two Lm strains of zoonotic origin, the Lm strain EGD and the Lm strain EGD-e (57/92, 61.9%), and with the Lm human strain 10403S (57/92, 61.9%), also belonging to the genetic lineage II.

**Table 3:** Feature differences between the Lm strain AUF and other Lm reference strains of zoonotic origin using BIGSdb-Lm database (https://bigsdb.pasteur.fr/listeria/).

The Lm Strain	Genome size (bp)	Comparison the number of genes/loci functionally annotated and associated with Lm					
		Virulence	Antibiotic Resistance	Metal Resistance*	SSI-1	Lm Genomic Islands	Motility
AUF	2,942,932	56	5	0	5	2	29
EGD	2,908,293	57	5	0	5	2	29
10403S	2,903,106	57	5	0	5	2	31
EGD-e	2,944,528	57	5	2	5	2	29
FDAARGOS 607	2,992,056	64	5	1	1	34	29
4/52-1953	2,973,626	41	5	0	1	2	29
UKVDL9	2,885,142	17	2	0	2	1	27
UKVDL7	2,929,607	15	1	0	1	2	27
UKVDL4	2,893,649	34	4	0	2	1	28
NTSN	2,904,500	62	5	0	1	2	29
FSL-J1-158	2,850,981	16	1	0	1	1	27

*cadmium resistance genes, *cadA* and *cadC*^25^.

Importantly, 14 specific loci were identified in both Lm strains of the lineage I, the Lm strain FDAARGOS_607 and the Lm strain NTSN (Table 1), but not in the Lm strain AUF^25^. There were the following genes: of LIPI-3, the additional sub-lineage pathogenicity island encoding listeriolysin S (LLS), a hemolytic toxin, a bacteriocin^31,32^ (LIPI3_llsB (LMOf2365_1116), LIPI3_llsD (LMOf2365_1118), LIPI3_llsY (LMOf2365_1117), LIPI3_llsG (LMOf2365_1113), LIPI3_llsH (LMOf2365_1114), LIPI3_llsP (LMOf2365_1119), LIPI3_llsX (LMOf2365_1115), LIPI3_llsA (LMOf2365_1112a); *srtB* (lmo2181), encoded the sortase B peptidoglycan-anchored protein induced in low iron conditions and involved with other sortases in many aspects of pathogenesis, from biofilm formation, adhesion, and immune suppression to bacterial loads and lethality^82,83^; *agrC* (lmo0050), responsible for the expression of AgrC, an integral membrane protein, a member of the class 10 receptor histidine protein kinases which are identified in Lm and involved in a quorum sensing system for the regulation of virulence (internalization, toxins) and biofilm^84–87^; *vip* (lmo0320), encoded vegetative insecticidal proteins (VIPs), toxins, during the vegetative growth phase by different bacterial species^88^; *gltB* (LMOf2365_2741), which encodes the major subunit of the glutamate synthase; *gltA* (LMOf2365_2740), citrate synthase gene, a critical mediator of site-specific fitness of pathogens during infection due to its influence on metabolic flexibility^89^; aut_IVb (LMOF2365_RS00075), which encodes autolysin, a member of a group of bacterial hydrolases involved in Lm invasion^90–92^. Less difference was noted between the Lm strain AUF and the Lm strain EGD. The latter strain contained only two additional loci, *srtB* (lmo2181) and *agrC* (lmo0050) which were absent in the Lm strain AUF, but were found in the fully virulent strains, such as the Lm strain EGD-e, the Lm strain NTSN, and the Lm strain FDAARGOS_607. We identified three loci, *srtB* (lmo2181), *agrC* (lmo0050), and *vip* (lmo0320) in the Lm strain EGD-e and in the Lm strain 10403S but not in the Lm strain AUF. The Lm strain NTSN had 13 additional loci which were not found in the Lm strain AUF. The same loci were present in the Lm strain FDAARGOS_607, except for aut_IVb (LMOF2365_RS00075), and absent in the Lm strain AUF. Only two specific loci (*agrA* (lmo0051) and *comK* (LMOf2365_2303)) were revealed in the Lm strain AUF, being absent in the Lm strain 10403S^25^. The locus *prfA* (lmo0200), which encodes the transcriptional factor PfrA, the master regulator of Lm major virulence genes^39,93–95^, was identified in the Lm strain AUF similarly with other Lm reference strains^25^. Remarkably, the Lm strain AUF PrfA demonstrated no 2 amino acid changes typical for the Lm strain EGD (Serine → Glycine, S145G; Tyrosine → Cysteine, Y229C), resulting in the constitutive overexpression in the Lm strain EGD of several major virulence genes^39^ although the marked *prfA* nucleotide sequence polymorphism^96^. Multiple sequence alignment of the Lm strains AUF PrfA protein versus other Lm reference strains showed its identity with those in the Lm strains, the Lm strain EGD-e, the Lm strain FDAAGROS_607, the Lm strain NTSN, the Lm strain UKVDL4 and the Lm strain 10403S^97^, thus supposing the possible expression in the Lm strain AUF of the PrfA-regulated virulence genes comparable to the Lm strain EGD-e and other virulent Lm strains as well. The locus *hly* (lmo0202) encoded the pore-forming toxin listeriolysin-O (LLO), allowing Lm to escapes the phagosome to avoid lysosomal killing^43,98^ was also identified in the Lm strain AUF and other Lm reference strains^96,99^. The alignment of the Lm strain AUF LLO protein was almost identical to those in the Lm strain EGD, the Lm strain EGD-e and the Lm strain 10403S, although differed from all the other Lm strains by a single amino acid substitution of one Serine to Lysine, S523K. Also, there were 3 other amino acid substitutions, namely: (i) Threonine to Asparagine, T309N in the Lm strain AUF and other strains except the Lm strain FSL-J1-158; (ii) Asparagine, N in position 31 in the Lm strain AUF, the Lm strain EGD, the Lm strain EGD-e, the Lm strain 10403S, the Lm strain 4/52-1953, the Lm strain FDAAGROS_607 and the Lm strain NTSN instead of either Histidine, H in the Lm strain UKVDL4, the Lm strain UKVDL7 and the Lm strain UKVDL9 or Glutamine, Q in the Lm strain FSL-J1-158; (iii) Serine, S in the position 35 in the Lm strain AUF, the Lm strain EGD, the Lm strain EGD-e, the Lm strain 10403S, the Lm strain 4/52-1953 and the Lm strain FSL-J1-158 instead of Leucine, L in the Lm strain FDAAGROS_607, the Lm strain NTSN, the Lm strain UKVDL4, the Lm strain UKVDL7 and the Lm strain UKVDL9^99^.

Also, 6 specific loci (*agrA* (lmo0051), *ami* (lmo2558), *aut* (lmo1076), *inlG* (lmo0262), *tagB* (lmo1088), and *inlL* (LMON_RS10535), were found only in the Lm strain AUF and were not identified in the Lm strain FDAARGOS_607, while only a single locus, *inlC* (lmo1786) was absent in the Lm strain EGD (locus identified in the Lm strain FDAARGOS_607). We found in the Lm strain AUF two loci, *inlD* (LMON_RS01345) and *comK* (LMOf2365_2303) that were not recognized in the Lm strain EGD-e (both of them were also identified in the Lm strain FDAARGOS_607). At least seven loci, *prsA2* (lmo2219), *fbpA* (lmo1829), *ami* (lmo2558), *aut* (lmo1076), *inlG* (lmo0262), *inlL* (LMON_RS10535), and *tagB* (lmo1088) were undetectable in the Lm strain NTSN strain while present in the Lm strain AUF^25^. There were 12 out of 56 (21.5%) identical loci present in the Lm strain AUF and the Lm strain FSL-J1-158 of the genetic lineage IV, and from 13 out of 56 (23.2%) to 39 out of 56 (69.6%) in the Lm strains of the genetic lineage III. Apparently, either all or some of these loci could be potentially either directly or indirectly involved in the mechanisms of residual virulence of the Lm strain AUF.

**Fig. 3:**
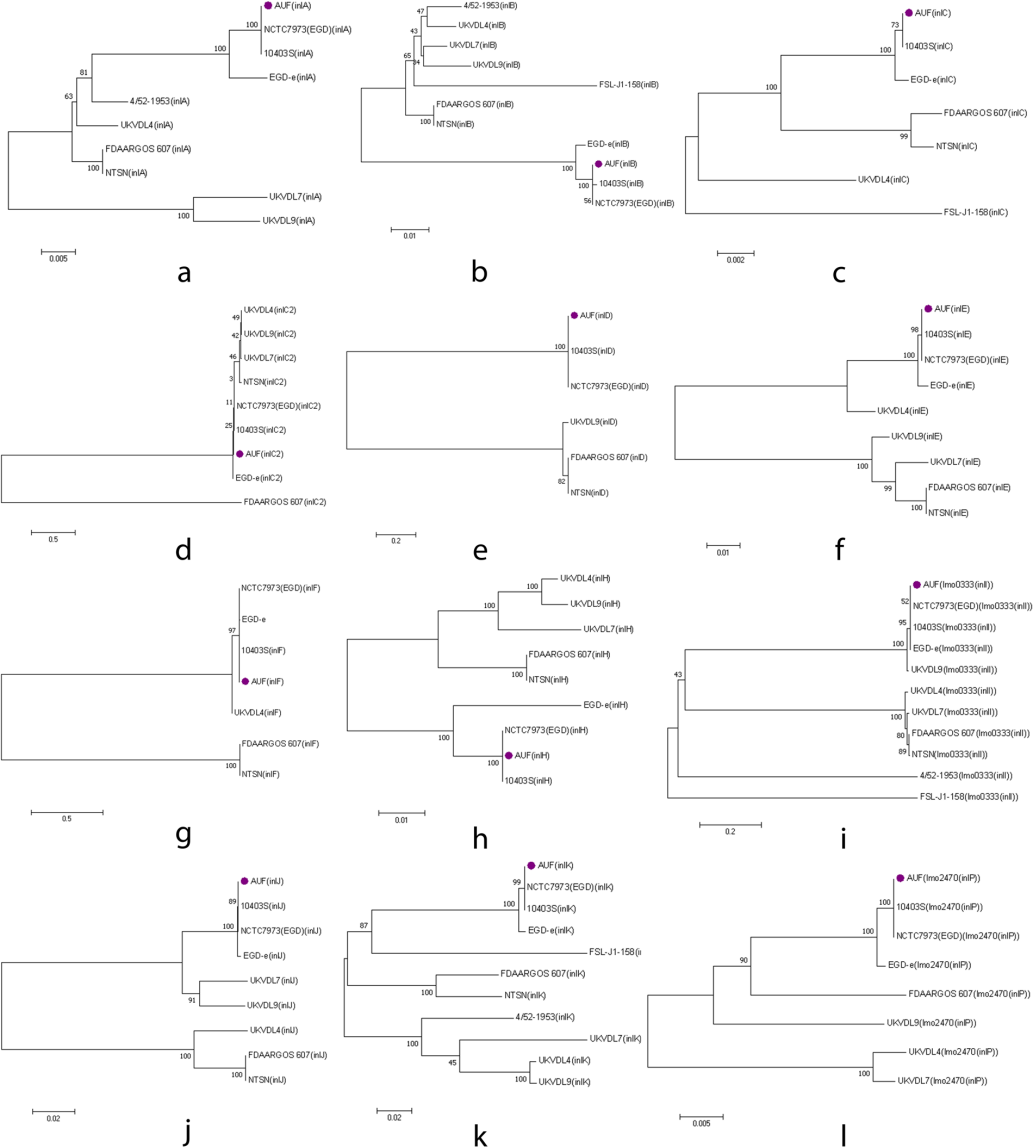
Phylogenetic analysis of the internalins in the Lm strain AUF and the reference Lm strains. (**a**) the Lm internalin A. (**b**) the Lm internalin B. (**c**) the Lm internalin C. (**d**) the Lm internalin C2. (**e**) the Lm internalin D. (**f**) the Lm internalin E. (**g**) the Lm internalin F. (**h**) the Lm internalin H. (**i**) the Lm internalin I. (**j**) the Lm internalin J. (**k**) the Lm internalin K. (**l**) the Lm internalin P. Phylogenetic trees were constructed using MEGA-7 (https://www.megasoftware.net/). The Bootstrap value is 100.

Furthermore, 14 genes encoding biosynthesis of members of the internalin multigene family^100^, internalins (*inlA* (lmo0433), *inlB* (lmo0434), *inlC* (lmo1786), *inlC2* (LMON_RS01340), *inlD* (LMON_RS01345), *inlE* (lmo0264), *inlF* (lmo0409), *inlG*, *inlH* (lmo0263), *inlI* (lmo0333), *inlJ* (lmo2821), *inlK* (lmo1290), *inll* and *inlP* (lmo2470) were found in the Lm strain AUF and the majority of the Lm strains belonging to the genetic lineages I-II, while rarely detected in the representatives of the genetic lineages III-IV (Table 4). Comparative analysis of the main internalin genetic characteristics showed that the majority of genes and the relevant allelic profiles of the Lm strain AUF were almost identical to those found in the Lm strain EGD and the Lm strain 10403S strains. The *inlC* was present in the Lm strain AUF and absent in the Lm strain EGD. The difference between the Lm strain AUF and the Lm strain 10403S was found only in the alleles for the *inlB*. Only a single identical allele (*inlG*) was present in both the Lm strain AUF and the Lm reference strain EGD-e. No identical allele profiles for these genes were found between the Lm strain AUF and both Lm strains of the genetic lineage I, the Lm strain FDAARGOS_607, and the Lm strain NTSN. The Lm strain AUF, likewise other Lm strains of the genetic lineage II, the Lm strain 10403S, the Lm strain EGD, and the Lm reference strain EGD-e, showed a certain diversity in internalin gene compositions versus the Lm strains of the genetic lineages III-IV, similarly to other reports^100–102^. No internalin genes, including *inlA* and *inlB*, in the form of either pseudogenes or truncated variants were present in the Lm strain AUF and the Lm strain 10403S, the Lm strain EGD, and the Lm strain EGD-e unlike the Lm reference strains of both these genetic lineages (Table 4). However, there was a marked polymorphism in the multiple sequence alignment of *inlA*^96,103^ and *inlB*^96,104^. The Lm strain AUF together with the Lm strain EGD and the Lm strain 10403S differed from other Lm reference strains by the presence of 3 major amino acid substitutions in InlA protein as T51A (Alanine instead of Threonine), S187N (Asparagine instead of Serine), and A594P (Proline instead of Alanine). Further, we found in the same protein of the Lm strain AUF and 3 other phylogenetically close Lm strains, the Lm strain EGD, the Lm strain EGD-e and the Lm strain 10403S at least 10 amino acid substitutions, such as R3K (Lysine instead of Arginine), S142T (Threonine instead of Serine), N474S (Serine instead of Asparagine), S476P (Proline instead of Serine), Y530H (Histidine instead of Tyrosine), T648S (Serine instead of Threonine), T664A (Alanine instead of Threonine), N738D (Aspartate instead of Asparagine), I781L (Leucine instead of Isoleucine) and V790M (Methionine instead of Valine)^97,103^. More differences were found for *inlB* alignments. The InlB amino acid sequences of the Lm strain AUF and both the Lm strain EGD and the Lm strain 10403S had a single amino acid substitution in position 396 resulting in a change of Alanine to Threonine (A396T). The Lm strain AUF demonstrated 23 either single or double substitutions as amino acid changes in the InlB identical to those found in three genetically related Lm strains, the Lm strain EGD, the Lm strain EGD-e and the Lm strain 10403S, which were not identified among other Lm reference strains^96,104^. There were such changes as: L176I (Isoleucine instead of Leucine), S205A (Alanine instead of Serine), S246P (Proline instead of Serine), T/M251S (Serine instead of Threonine/Methionine), I262T (Threonine instead of Isoleucine), I291T (Threonine instead of Isoleucine), S373N (Asparagine instead of Serine), M387V (Valine instead of Methionine), E446K (Lysine instead of Glutamate), I479M (Methionine instead Isoleucine), I483R (Arginine instead Isoleucine), P486S (Serine instead Proline), A489S (Serine instead of Alanine), TL501-502KH (Lysine-Histidine instead of Threonine-Leucine), D533G (Glycine instead of Aspartate), I555K (Lysine instead of Isoleucine), IQ558-559TR (Threonine-Arginine instead of Isoleucine-Glutamine), GN568-569AG (Alanine-Glycine instead of Glycine-Asparagine), V578A (Alanine instead of Valine), S580N (Asparagine instead of Serine), W584R (Arginine instead of Tryptophan), T594K (Lysine instead of Threonine), RT599-600CQ (Cysteine-Glutamine instead of Arginine-Threonine). As expected, phylogenetically, all the internalins of the Lm strain AUF formed a single branch with only the Lm strain EGD and the Lm strain 10403S (Fig. 3a,b,e,f,h,j,k,l), being additionally clustered with the Lm strain EGD-e (Fig. 3c,d) but not with other Lm strains (Fig. 3a-l). Moreover, in the Lm strain AUF some of the internalins, *inlA*, *inlB*, *inlC2*, *inlD*, *inlE* and *inlG* were found as internalin gene clusters^105^, inlAB and inlC2DEG, while others, *inlC*, *inlF*, *inlH*, *inlI*, *inlJ*, *inlK*, *inlL* and *inlP* were located on the chromosome outside any cluster (Fig. 4.1) similarly with the majority of the Lm reference strains (Fig. 4.2, 4.3, 4.4, 4.5, 4.6, 4.7, 4.8, 4.9, 4.10, 4.11). Two identical internalin gene clusters were identified in the Lm strain EGD (Fig. 4.3) and in the Lm strain 10403S (Fig. 4.11). The internalin gene cluster inlAB relevant to the Lm strain AUF was also revealed in the Lm strain EGD-e and almost all the Lm reference strains (Fig. 4.4, 4.5, 4.7, 4.9, 4.10) except the Lm strain UKVDL7 (Fig. 4.8). The second internalin cluster inlC2DEG identified in the Lm strain AUF, the Lm strain EGD and in the Lm strain 10403S was only partially presented in other Lm reference strains as either inlC2DE in the Lm strain NTSN (Fig. 4.5) or inlDH in the Lm strain FDAARGOS_607 (Fig. 4.4), or inlEH in the Lm strain UKVDL4 (Fig. 4.7), or the Lm strain UKVDL7 (Fig. 4.8), or inlDEH in the Lm strain UKVDL9 (Fig. 4.9). The position of both internalin gene clusters was on the relevant Lm chromosome region on about 2.0 Mbp (Fig. 4.1), while for the Lm strain EGD-e, the Lm strain EGD, the Lm strain NTSN, the Lm strain UKVDL4, the Lm strain UKVDL9 and the Lm strain 10403S these loci were found within 0.3 – 0.5 Mbp (Fig. 4.2, 4.3, 4.5, 4.7, 4.9, 4.11) or about 0.9 – 1.1. Mbp for the Lm strain FDAARGOS_607 (Fig. 4.4), or 0.5 Mbp for the Lm strain FSL-J1-158 and the Lm strain 4/52-1953 (Fig. 4.6, 4.10). Probably, some of these genes can encode for the products potentially involved in the “residual virulence” of the Lm strain AUF, resulting in occasional vaccine-related adverse effects in vaccinated animals.

**Table 4:** Characterization of allelic profiles of genes encoding the biosynthesis of internalins identified in the genomes of the Lm strain AUF and the Lm reference strains isolated from animals with listeriosis using BIGSdb-Lm database (https://bigsdb.pasteur.fr/listeria/).

The Lm Strain	Allele													
	*inlA*	*inlB*	*inlC*	*inlC2*	*inlD*	*inlE*	*inlF*	*inlG*	*inlH*	*inlI*	*inlJ*	*inlK*	*inlL*	*inlP*
AUF	2	2	2	1	1	2	2	1	2	21	2	2	1	7
EGD	2	2	—	1	1	2	2	1	2	21	2	2	1	7
10403S	2	23	2	1	1	2	2	1	2	21	2	2	1	7
EGD-e	1	1	1	3	—	1	1	1	1	1	1	1	2	1
FDAARGOS_607	3	4	8	2	3	5	6	—	4	182	168	35	—	4
FSL-J1-158	—	369	18	—	—	—	—	—	—	78* (3752 SNP), P**	—	23* (20 SNP)	—	—
NTSN	3	4	6	2	3	5	6	—	4	4	168	5	—	—
UKVDL9	992* (24 SNP)	719* (16 SNP)	292* (2 SNP)	—	248* (2 SNP)	226	—	—	346* (2 SNP)	365* (85 SNP)	405* (6 SNP)	78* (14 SNP)	—	5578 (3 SNP)
UKVDL4	917	700	122	236* (12 SNP)	—	255 (19 SNP)	391* (19 SNP)	—	290* (12 SNP)	363	377	78* (1 SNP)	—	95
4/52—1953	30	27	—	—	—	—	—	—	—	135* (3745 SNP) псевдо**	—	24	—	—
UKVDL7	339	435* (17 SNP)***	—	244* (18 SNP)	—	267* (8 SNP)	—	—	298* (18 SNP)	400* (61 SNP)	466* (32 SNP)	78* (213 SNP)***	—	158* (4 SNP)

The numbers in the cells are the allele numbers relevant for each of the Lm strain. The numbers in the brackets are the numbers of SNPs in the nucleotide sequence of the individual gene of the relevant Lm strain. *the closest match profile in the BIGSdb-Lm database (https://bigsdb.pasteur.fr/listeria/); **pseudogene, P; ***The last 3- 5 amino acids are truncated in the relevant sequence.

**Fig. 4:**
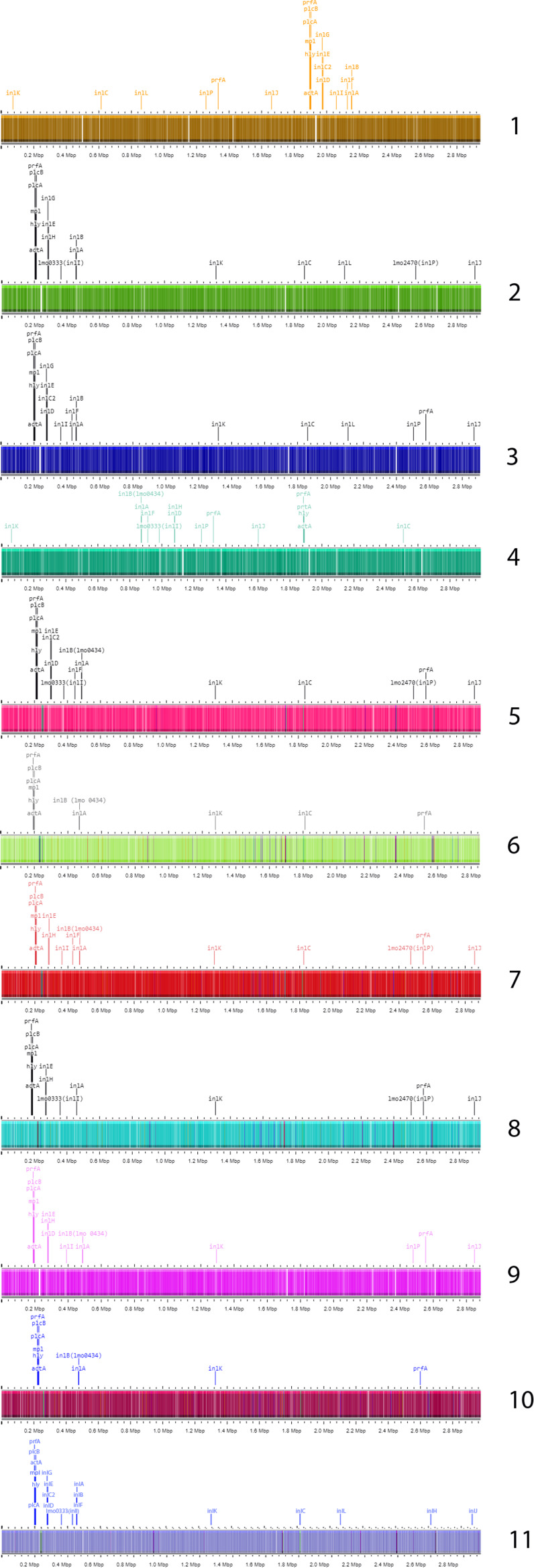
Linear maps of chromosomes of 10 Lm strains indicating virulence genes, including internalins. (1) The Lm strain AUF. (2) The Lm strain EGD-e. (3) The Lm strain EGD. (4) The Lm strain FDAARGOS_607. (5) The Lm strain NTSN. (6) The Lm strain FSL-J1-158. (7) The Lm strain UKVDL4. (8) The Lm strain UKVDL7. (9) The Lm strain UKVDL9. (10) The Lm strain 4/52-1953. (11) The Lm strain 10403S. Vertical bars reflect CDSs.

Five antibiotic resistance genes were annotated in the Lm strain AUF related to *Listeria* antibiotic resistance (*fosX* (lmo1702), fosfomycins^106^; *mprF* (lmo1695), cationic antimicrobial peptides^107^; *lin* (lmo0919), lincosamides^108^; *norB* (lmo2818), quinolones^109^; and *sul* (lmo0224), sulfonamides^109^), which were similar to those existing in two fully virulent Lm strains of the lineage I, the Lm strain FDAARGOS_607 and the Lm strain NTSN, and both homologs, the Lm strain EGD, the Lm strain EGD-e and the Lm strain 10403S (the lineage II), as well as in the Lm strain 4/52-1953 isolate of the genetic lineage III (Table 3). Four of the same five genes, *fosX*, *mprF*, *lin* and *sul*, were found in the Lm strain UKVDL4. Only some of these genes were annotated in other Lm strains of the lineages III-IV, the Lm strain UKVDL7 (n = 1), the Lm strain UKVDL9 (n = 2), and the Lm strain FSL-J1-158 (n = 1)^25^.

No metal resistance genes were found in the Lm strain AUF using BIGSdb-Lm database (https://bigsdb.pasteur.fr/listeria/)^25^. Similarly, no relevant genes were revealed in the majority of other Lm reference strains while cadmium resistance genes, *cadA* and *cadC* ^110,111^, were identified in the Lm strain EGD-e and the Lm strain FDAARGOS_607 (Table 3)^25^. We found no significant difference in the number of genes involved in motility in the Lm strain AUF compared with other Lm strains. However, the number of Lm Genomic Islands-associated genes found in the Lm strain AUF (n = 2) was markedly (18 times) lesser those present exclusively in the Lm strain FDAAGROS_607 (n = 36).

Quantitatively, the number of genes of SSI-1 (n = 5) in the Lm strain AUF strain was the same only with the closest homologs, the Lm strain EGD, the Lm strain EGD-e and the Lm strain 10403S, and exceeded those which were annotated for other reference strains independently from their genetic lineage, including both fully virulent strains, the Lm strain FDAARGOS_607, and the Lm strain NTSN, by 2.5 - 5 times. In contrast to the Lm strain AUF, only a single gene, SSI1_lmo0444 (lmo0444), was found in the Lm strain FDAARGOS_607, the Lm strain 4/52-1953, the Lm strain UKVDL7, the Lm strain NTSN and the Lm strain FSL-J1-158. Two genes of the SSI-2, associated in Lm with a tolerance to alkaline and oxidative stresses, SSI2_lin0464 (lin0464) and SSI2_lin0465 (lin0465)^112^ and absent in the Lm strain AUF, were revealed in the Lm strain UKVDL4 and the Lm strain UKVDL9^25^.

Importantly, we found pronounced polymorphisms in the majority of the alleles of the genes responsible for antibiotic resistance and motility, SSI-1 and determination of Lm Genomic Islands in the Lm strain AUF compared with the other Lm strains used^25^. The Lm strain AUF demonstrated almost total identity exclusively with only the Lm strain EGD and the Lm strain 10403S but not with other Lm strains independently from their genetic lineage, ST, and CC.

In order to examine the specific contribution of UVR to the inactivation of the Lm strain AUF genes, we compared all the 17 relevant pseudogenes found in the genome of this strain with other Lm genomes available in GenBank using the BLAST resource (https://blast.ncbi.nlm.nih.gov/Blast.cgi). We identified only four putative genes, *No. 2* (locus tag: GZH80_03675), *No. 4* (locus tag: GZH80_06950), *No. 6* (locus tag: GZH80_08750), and *No. 13* (GZH80_11730), which were assigned in the Lm strain AUF as pseudogenes, while in the majority of other Lm strains (up to 99-100 of 100 strains available) were annotated as genes encoding functional protein products^113^. These proteins were the following: (i) 100 out 100 Lm strains: tetratricopeptide repeat protein, sensor histidine kinase; and amino acid permease, and (ii) 99 out of 100 Lm strains: a hypothetical protein which was found as the pseudogene with 100% identity to the Lm strain AUF in only a single Lm strain 3453. The relevant genes demonstrated homology on the level of 92.87 – 99.93% versus the Lm strain AUF as a result of the presence of either frameshift mutations in genes No. 2, 6, and 13, or missing C-terminus in the gene *No. 4*. The products expressed by these genes are involved in bacterial pathogenesis by controlling the expression of virulence, biofilm formation, protein-protein interactions, antimicrobial resistance, quorum sensing and signaling^27,114–116^. The relevant mutants in wild Lm strains have been reported to be defective for intracellular growth and cell-to-cell spread and were severely attenuated for virulence in a mouse model^114^. At the same time, when the genome of the Lm strain AUF was compared with the genomes of its closest homolog, the Lm strain EGD, we found only a single pseudogene (1/17 representation in the Lm strain AUF), *No. 9* (LMON_0171), with possible expression of only a truncated hypothetical protein^113^. Furthermore, in the genome of the Lm strain 10403S four of 17 pseudogenes related to those in the Lm strain AUF^78^ due to frameshift mutations were annotated, such as: *No. 7* (LMRG_02848), *No. 11* (LMRG_00154), *No. 12* (LMRG_00322) and *No. 15* (LMRG_02951). We believe some of these genes could be damaged by UVR resulting in the UVR-induced DNA mutations leading to the Lm strain AUF attenuation.

## Usage Notes

Currently, we present the complete genome sequence and the gene annotations for the Lm strain AUF, which has been used for decades as a live whole-cell veterinary vaccine. We compared the Lm strain AUF genome with the whole genomes of reference fully virulent Lm strains, of the Lm isolates derived from animals with listeriosis, and the Lm strain used for the development of live attenuated vaccine vectors against cancer. We believe that the data obtained will be useful to unravel the mechanisms of attenuation and virulence in *Listeria* and other pathogenic microorganisms. However, it is very difficult to make objective conclusions on the Lm strain AUF attenuation in the absence of the wild parental Lm strain ‘A’. Nevertheless, we hope that these data will be valuable as the basic platform for development of the effective and safe new-generation vaccine(s) with improved characteristics for prophylaxis of listeriosis in animals worldwide .

**Table 5:** Versions of software and database used in this study.

Software/Database	Application	Version/Date
AfterQC	Automatic filtering, trimming, error removing and quality control for fastq data from Illumina HiSeq 2500	v0.9.7
Filtlong	Filtering long reads by quality	v0.2.1
Porechop	Adapter trimmer for Oxford Nanopore reads	v0.2.4
Unicycler	Genome assembly	v 0.4.9
PGAP	Annotation of genome	v 4.11
BIGSdb	Identification of virulence genes and MLST typing	v 1.42.0
CARD database	Antibiotic resistance genes prediction	v 6.0.2
MEGA 7	Phylogenetic analysis	v 7.0.26
REALPY	Whole genome phylogenetic analysis	v 1.13
Proksee	Genome map visualization	Jun 2023
BV-BRC	Proteome Comparison	v 3.30.19

### Supplementary information

Supplementary Table 1

Supplementary Table 2

Supplementary Table 3

Supplementary Table 4

## Data Availability

Software used in the generation or processing of our data is stated in the Methods section. Detailed information including versions of software and database are provided in Table 5.
